# Quantitative datasets of societal value, technology and policy for human-water system modelling

**DOI:** 10.1038/s41597-025-05885-x

**Published:** 2025-09-01

**Authors:** Shuanglei Wu, Yongping Wei, Jing Wei, Yonglan Xiong, Sarina Huang, Ratri Werdiningtyas, Paulina Genova, Paul Hong, Lin Gan, Chendi Song

**Affiliations:** 1 School of Public Policy and Management, Guangxi University, Nanning, 530004 China; 2 School of the Environment, the University of Queensland, Brisbane, 4072 Australia; 3 Department of Hydraulic Engineering, State Key Laboratory of Hydroscience and Engineering, Tsinghua University, Beijing, 100084 China; 4 National Science Library (Chengdu), Chinese Academy of Science, Chengdu, 610213 China; 5 Room 1704, Building A, Fenglan GuoJi Mall, 32 Xizhimen Street North, Beijing, 100082 China; 6 Urban and Regional Planning Study Program, Faculty of Engineering, Universitas Sebelas Maret, Ir. Sutami 36 A Kentingan, Surakarta, 57126 Indonesia; 7 WSP Chile and Argentina, Santiago, 7650784 Chile

**Keywords:** Environmental social sciences, Water resources

## Abstract

Value, technology, and policy are three interactive societal factors affecting the willingness, capacity, and formal rules of human interactions with water. Existing human-water models generally neglect the dynamic and accumulated processes of these factors, failing to explain the societal causes of changes in water practices. Here we developed nine process-based quantitative datasets of Value-Technology-Policy regarding water. They contain 17,003 newspaper articles and 801 public submissions to reflect different water values, 1337 ancient technologies and 40,303 patents for water technologies, and 720 water policy documents at various spatial (national, regional, state, and river basin) and temporal (decades to hundreds of years) scales. A consistent, 4-step content analysis approach was adopted to identify, collect and manually code five key elements (processes) of value, technology and policy: the time (“when”), location (“where”), actor (“who”), theme (“what”), and perspective/tone (“what effects”). Inter-coder reliability tests were conducted to ensure the consistency and validity of the data. These datasets will contribute to more process-oriented understanding of societal factors for improved human-water system modelling in the Anthropocene.

## Background & Summary

Water is a fundamental natural element that links and supports the cycling of nutrients, energy, and carbon in the Earth System, and a critical resource at the core of the continued prosperity of the human society for food security, industrial development, and urbanization^1^. The emergence of the Anthropocene has marked the end of hydrological stationarity as a result of human activities^2,3^, making humans an integral part of the hydrological system rather than exogeneous drivers^4,5^.

Humans interact with water through their societal practices on water supply, water use and water management. Value, technology, and policy are three interactive and essential societal factors affecting human interactions with water. Values are a set of opinions, beliefs, norms, and attitudes shared by the majority of a regional population, which determine the human willingness of change in their water practices^6,7^. Technologies permeate all aspects of human lives, powering or constraining the interactions via changing the capacities of water utilizations^8,9^. Policies are the formal rules set by governments to regulate water supply and use^10,11^. Values affect the public’s choices of technologies and policy development by changing the expectation regarding water resources, and the implementations of policies and technologies in turn open new possibilities for changes in water values^12,13^. Over time, value, technology, policy and their interactions explain human development in the broadest sense and act as drivers for intended and unintended changes in the natural processes, forming highly complex, non-linear human-water systems.

There have been vibrant development of conceptual frameworks and models including coupled human-natural systems (CHNS)^14^, socio-ecological systems (SES)^15^, and socio-hydrology^16^ for simulating and predicting the complex human-water relationships. The “big data revolution” has enabled accesses to large online textual archives, providing “thick” descriptions of societal phenomena including value, technology and policy development for social science studies. Compared with quantitative data, texts (qualitative data) pose huge challenges for integrating them into quantitative analysis (e.g. hydrological modelling). The common approach adopted in the existing human-water system models is to use proxy indicators, for example using the level of insurance coverage to represent level of flood risk awareness^17^, using income and water price to represent water use behaviours^18^, and using levee height to represent technological flood protection capacity^19^. However, the singular representation of proxy indicators neglected the complex and dynamic processes of societal factors: what happened, who were involved, under what contexts, what were different perspectives on what happened, and what were the outcomes? All these processes, although highly descriptive, are necessary elements representing societal factors. Failing to link these processes in the human-water system models will result in failure to explain the societal causes of changes in water practices. Therefore, developing process-based datasets for quantifying the societal factors (value, technology and policy) from text archives regarding water will provide a more systemic representation of these factors, enabling formalization and incorporation of them into human-water system models.

To develop process-based datasets, we first adopted Harold Lasswell’s communication theory to systemically and consistently extract qualitative narratives of the three societal factors (value, technology and policy) regarding water from different textual sources. American sociologist Harold Lasswell in his book *The Structure and Function of Communication in Society* developed a “5 W” model containing five fundamental elements: Who, What, In Which Channel, To Whom, and With What Effect^20^. These questions represent the most salient elements of communication. As one of the earliest and most influential communication models, the “5 W” model has been widely used as a starting point by many theorists for the development of their own theories applying to specific fields^21–23^. Lasswell himself also expanded this model to “7 W” to reflect medias’ influences on policy decisions: (1) Participants: the people and organizations with a stake; (2) Perspectives: the varied viewpoints of these stakeholders; (3) Situations: where the stakeholders interact; (4) Base Values: assets used by stakeholders to pursue their goals (power, wealth, skills etc.); (5) Strategies: approaches used by stakeholders to achieve their goals, (6) the short term “outcomes” and (7) long-term “effects”^24^. Taking into consideration of the requirement of developing human-water models, the nature of the societal factors and of different data sources, our datasets adjusted the contents and numbers of Ws collected for each of the value, technology, and policy factors. For value, the perspective/sentiment expressed by people was coded as an important element reflecting people’s value regarding a water issue to replace “What Effect”. For technology and policy, “What Effect” was not included as the information was not documented in the data sources. More details can be found in the Methods and the Supplementary Information sections.

We then adopted content analysis^25^, an approach commonly used in many social science studies, to transform the extracted qualitative narratives of three societal factors (value, technology and policy regarding water) from different textual sources into quantitative data. With a root in the Grounded Theory^26^, content analysis assumes that interpretations are “grounded in” observed empirical data. It follows specific coding techniques to classify and categorize text segments into a set of concepts, categories (constructs), and relationships, forming a context–mechanism–outcome configuration^27^. A codebook was created to guide the content coding process, which contained detailed description of each “W” in the communication model. An inductive coding approach that iteratively included additional contents for each element was used. Human coders manually coded all textual data collected, as it was believed that important meanings in texts were often implied, and that human coders were more alert to the implied and contextual meaning beneath the manifest content compared with computer coding, which typically extracted contents based on word appearance frequencies at the document level^28^. Using this time-intensive manual coding ensured that the datasets developed can be used to develop, calibrate and validate human-water system models^29^.

Finally, we adopted a long-time perspective to develop these datasets. As societal factors tend to have slow-changing characteristics^30^, and it is the interaction of the “fast” natural factors and the “slow” societal factors that determine the human-water system threshold which, if crossed, causes the system to move into a new state^4,31^. All data were thus collected from the longest possible timeframe, except where targeted research foci applied.

We developed nine datasets on three fundamental societal factors (value, technology and policy) regarding water. These datasets contain 17,003 newspaper articles, 801 public submissions, 1337 ancient technologies, 40,303 patents, and 720 policy documents in various spatial (national, regional, state, and river basin) and temporal (decades to hundreds of years) scales. They were extracted from either authoritative or widely used text archives (Tables 1–3).

**Table 1:** List of societal value datasets regarding water.

Societal factors	Data	“5 W” elements	Sampled?	Data periods	Data sources	No. of items	References for detailed data
Value	Evolution of societal value on water in Australia	“When”: Publication date “Where”: Publication location “Who”: Actors involved “What”: Themes “What effect”: Perspectives/Tones	Yes	1843–2020	Trove (1843–1954), The Sydney Morning Herald’s archives (1955–1986), Factiva (1987–2020) for news published by The Sydney Morning Herald	3,644	^51–53,58^
	Evolution of societal value on water in China	“When”: Publication date “Where”: Related locations “Who”: Actors involved “What”: Themes “What effect”: Perspectives/Tones	Yes	1946–2017	Factiva for news published by The People’s Daily	2,059	^52,54,59^
	The international public’s opinions on Three Gorges Dam in China	“When”: Publication date “Where”: Related locations “Who”: Actors involved “What”: Themes “What effect”: Perspectives/Tones	Yes	1982–2015	Factiva for 8 dominant newspaper from UK (The Times, The Guardian), USA (The New York Times, The Washington Post), Singapore (Lianhe Zaobao, The Straits Times), and Australia (The Australian, The Sydney Morning Herald)	267	^60^
	The public’s opinions changes from the two large flooding in Brisbane River, Australia	“When”: Publication date “Where”: Related locations “Who”: Actors involved “What”: Themes “What effect”: Perspectives/Tones	No	2011–2022	Factiva, covering 332 national, regional and local newspapers published in Australia.	11,033	N/A
	Stakeholders’ opinion on the water reform in the Murray Darling Basin in Australia	“When”: Publication date “Where”: Related locations “Who”: Actors involved “What”: Themes “What effect”: Perspectives/Tones	No	2015, 2017, 2019	Public submission repositories hosted by the Parliament of Australia, the Murray-Darling Basin Authority, and the New South Wales Department of Planning, Industry and Environment	801	^61^

**Table 2:** List of technology datasets regarding water.

Societal factors	Data	“5 W” elements	Sampled?	Data periods	Data sources	No. of items	References for detailed data
Technology	Water and agricultural technologies in ancient China	“When”: Application period “Where”: Application locations “Who”: Actors involved “What”: Names and types of technologies	No	8000 BC – 1912 AD, divided into 8 historical periods based on dynastic cycling	Historical encyclopaedias: The Development History of Chinese Agriculture; The History of Science and Technology in China – The Agriculture Volume; The History of Chinese Agricultural Technologies	1337	^62–65^
	Global river-related patents	“When”: Application date “Where”: Registration location “Who”: Patent applications “What”: Themes	No	1900–2020	Pantentscope administered by the World Intellectual Property Organization (WIPO)	40,303	^66,67^

**Table 3:** List of policy datasets regarding water.

Societal factors	Data	“5 W” elements	Sampled?	Data periods	Data sources	No. of items	References for detailed data
Policy	Water regulations in Victoria, Australia	“When”: Publication date “Where”: Related locations “Who”: Actors involved “What”: Themes	No	1862–2016	Australian legal repository: Australasian Legal Information Institute (AUSTLII)	425	^68^
	Water regulations in Chile	“When”: Publication date “Where”: Related locations “Who”: Actors involved “What”: Themes	No	1900–2019	Chilean legal repository: Ley Chile	295	^69,70^

**Fig. 1:**
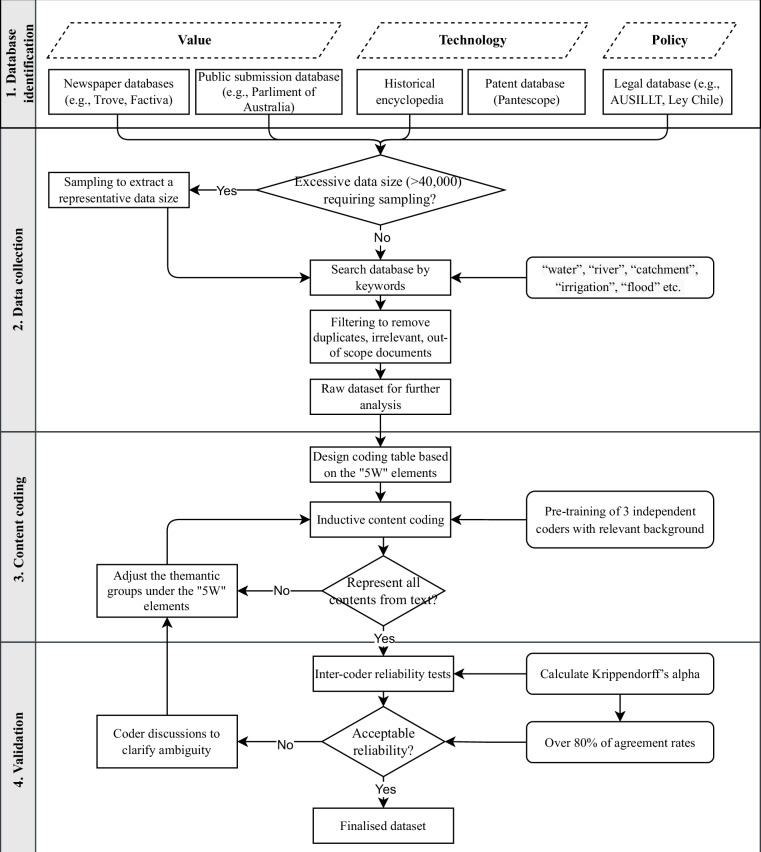
Workflow of data processing.

Four steps were followed to generate the datasets (Fig. 1). First, data sources on value, technology, and policy regarding water were identified. These data sources are authoritative and representative online archives with long-time data available, including the global news archives, governmental document repository, historical encyclopaedias, the World Intellectual Property Organization database, and national policy databases. Second, textual documents archived in these data sources were searched by designed keywords. If an excessive number of textual documents were returned from the search (e.g., newspaper articles with daily publications for over 100 years exceeding 40,000 articles), these documents were sampled to create a representative subset manageable for manual content analysis. Search keywords were designed to ensure documents collected had targeted focus on the research topic, and filtering was then conducted to remove duplicates, irrelevant, and out-of-scope documents. In the third step, a coding framework was consistently applied to all documents with five key elements of value, technology, and policy where possible. The five elements (referred to as “5Ws” thereafter) are the time (“when”), location (“where”), actor (“who”), theme (“what”), and perspective/tone (“what effects”), which together define the developmental processes of these societal factors. Finally, inter-coder reliability tests were conducted to ensure the consistency and validity of the coding results.

These datasets have potentially wide applications. First, these datasets can be used independently for clarifying goals, analysing local contexts, examining relationships between the “5Ws”, and identifying trends and patterns of value, technology, and policy related to water resource governance. This will allow determining and evaluating alternatives for future governance in their respective spatial contexts (China, Australia, and Chile). Second, they provide a starting point to integrate various datasets for comparisons and generating general insights on the three factors and their interactions across different case studies. This will allow developing systemic understanding of the societal drivers for intended and unintended changes in the complex human-water systems. Finally, these process-based quantitative datasets provide the first-hand data for development, calibration and validation of the human-water system models^32^. This improved modelling capacity will allow better prediction of the human-water relationships in the Anthropocene. Finally, we recognise that the data collected in these datasets only represent dominant forms of value, technology, and policy regarding water.

## Methods

We applied a consistent, systematic content analysis approach to collect and transform textual information into quantifiable data that represented value, technology, and policy development regarding water. The detailed steps are as follows:

### Data sources identification

#### Value

Societal values regarding water are a set of opinions, beliefs, norms, and attitudes shared by the majority of a regional population, which determine the human willingness of change in their water practices^6^. In these datasets, newspapers and public submissions were used to represent the development of societal values. Both data types provide long-term, traceable documentation of public interests and have been a crucial media to reflect societal opinions regarding water.

“It is the media that creates the reality and sets the public agenda^33^.” Newspapers provide an accessible, in-depth representation of topics reported to the public over a long timeframe. They shape and are shaped by public opinions according to the agenda setting theory, reflecting and deliberately framing the people and organizations involved, the situations in which they interact, and their perspectives^28^. While digital social media (e.g., Twitter, TikTok) have been increasingly used as platforms to reflect societal values, newspapers remain a dominant form of communication as people still consider them more authoritative and trust-worthy, thus reflecting the majority's values^34^. Likewise, leading newspapers with high circulation and broad readerships should be targeted to ensure sufficient representation of the development processes of values in a long time, and not geographically confined in terms of readerships^35^. Multiple online news archives, mainly Factiva and Trove were used as the data sources to collect historical newspapers.

Public submission documents are written comments submitted online or via mail regarding certain government initiatives. They have been one of the most common practices in social licensing procedures and used by government agencies to seek public participation globally^36^. Among other forms of public participation such as public hearings, open meetings, oral testimony, survey and interviews, these written comments submitted online or mailed in are directly sourced from people and organizations to reflect their background and sentiment on specific governance issues. These documents provide clear and authentic reflections of stakeholder inputs. They avoid potential bias or influences imparted from consultation reports prepared for the governing authorities. All submissions were retrieved from online repositories hosted by the receiving government agencies, including the Parliament of Australia, the Murray-Darling Basin Authority, and the News South Wales Department of Planning, Industry and Environment in Australia (Table 1).

#### Technology

Technological development co-exists with human history and is the major mean through which water is utilised. Technologies are broadly defined as the artefacts, methods, and practices “to fulfil certain human purposes in a specifiable and reproducible way”^37^. While data related to technological innovation have been readily available in the modern era (e.g., patents, scientific publications, research expenditures), historical technological development over a long timeframe is more difficult to retrieve yet has important value for modelling historical socio-hydrological phenomenon. In these datasets, historical encyclopaedias were selected as data sources to represent historical technological development, whereas patent documents were selected to represent modern technological development.

Historical encyclopedias integrate first-hand knowledge from ancient literature and excavations from archaeological discoveries. Compared with ancient literature and archaeological excavations, these documents are more accessible, less event-specific, and organized in a systematic, chronical manner covering the full duration of historical technological development. Encyclopaedias used for this dataset included: *The History of Chinese Agricultural Technologies* edited by Liang^38^, *The History of Science and Technology in China - The Agriculture Chapter* edited by Dong and Fan^39^, and *The Development History of Chinese Agriculture* edited by Yan and Yin^40^. In particular, the encyclopaedia edited by Liang^38^ and Dong and Fan^39^ highlighted in details the diverse spectrum of different technologies, while Yan and Yin^40^ focused on the co-development of technology and society. The *History of Science and Technology in China* edited by Dong and Fan is currently the most up-to-date and comprehensive encyclopedia in China about technologies, building from the renowned *Science and Civilization of China* by Joseph Needham^39,41^. All three encyclopedias were compiled by nation-wide research teams of archaeologists, historians, and sociologists, published by top Chinese publishing houses, and have been widely cited by the Chinese Agricultural Yearbooks^42,43^ and academic researchers^44^. Using all three encyclopaedias allows cross-validation of technologies documented, and a thorough coverage of the societal contexts, actors, and processes involved in their development.

For modern technologies, patents as one of the most common representatives of technology were used^45^. There are three major patent data sources globally: the Pantentscope (www.wipo.int/patentscope) administered by the World Intellectual Property Organization (WIPO), the Espacenet (www.espacenet.com) administered by the European Patent Office (EPO), and the U.S.PTO (https://www.uspto.gov/patents/search) administered by the United States Patent and Trademark Office (USPTO). Compared with the other two, Patentsocpe was selected in this dataset because it has comparatively higher filing standards, and focuses on global patent applications rather than those with local or country-specific focuses^46,47^. It stores more than 107 million patent documents from 193 countries, regions, and organizations dated back to 1782, providing full patent document texts on the specific technologies, the dates and locations of application and follows a consistent International Patent Classification (IPC) system to allow in-depth analysis of technologies over a long timeframe (Table 2).

#### Policy

Policies are formal institutions including laws, acts and regulations that determine who are at stake to make decisions and under what processes should the resources be used^48^. The policy documents can be seen as intentional abstract representations of formal governance that should be followed by managing authorities and resource users^49^. Different countries have different policy development frameworks. For example in Australia, acts form the highest level of legislation and other subordinate legislations are built based on acts. In Chile, formal regulations are sets of documents contained in legal frameworks called Laws, Decree-Law, Decree with Force of Law, Decrees and Resolutions. In these datasets, these policy documents were collected from official governmental repositories. The Australian Legal Information Institute (AUSTLII) (www.austlii.edu.au) contains all legislations and case laws ever enacted in Australia across all States, Territory, and the Commonwealth of Australia as early as the 1800s. Similarly, the Ley Chile (www.LeyChile.cl) data source is the most comprehensive archives storing over 245,000 policies in Chile since 1900 (Table 3).

### Data collection

#### Sampling

This is an optional step applying only to some newspapers with daily publications over a long timeframe to ensure they were manageable for manual content analysis. Four weeks of newspaper articles from each year were sampled with two methods proposed by Lacy, *et al*.^50^ and have been consistently applied in former publications^51–54^. The four weeks contain two constructed weeks, which accounted for the cyclic reporting of news from Monday to Sunday and were randomly selected from the whole year; and two consecutive weeks accounted for the short-term events (e.g., the Australian Water Week). One constructed week and one consecutive week of news articles were collected in each half year. These sampling methods have been shown to reliably account for a full year’s news contents^50,55^.

#### Filtering

All text documents collected from the specified archives were stored either as word documents or searchable PDF files. They were screened individually by the authors. Duplicate documents were first removed. Documents containing only incidental mentions of the specified keywords with no direct relevance to the issue of focus were then removed. Finally, documents concerning issues outside the spatial scope of interest were also removed.

### Content analysis

#### Coding table

We adopted a consistent coding framework based on the “5 W” model, which defined five key elements in the communication processes: “When”, “Where”, “What” happened, involving “Who” and leading to “What effect (perspective/tone)” (Table 4). A full list of contents under each element for each dataset can be found in the Supplementary Information.

**Table 4:** A “5 W” coding table for content coding.

“5 W” element	Description
“When”: Time	The time when the news article or policy document was published, or when the technology was invented, developed or implemented.
“Where”: Location	The spatial location associated with the news article, policy document, or the technology.
“Who”: Actor	The individual or organizations interested, involved, or affected by the news reporting, policy, or technology, can be broadly divided into two groups: the managing authorities and the users.
“What”: Theme	Topics of discussion deduced from text, such as agriculture, water quality and health, water engineering, terrestrial ecosystem.
“What effect”: Perspective/Tone	The perspectives or sematic tones expressed in the article, which is classified into the environmental-oriented and economic-oriented dimensions; or semantically positive, negative, and neutral.

The first and second elements provided information on the temporal and spatial scope of the values reflected in news articles, technologies and policies respectively. These two elements express “when” and “where” the developmental processes of the societal factors took place. The third element contained the actors involved in the news reporting, policy, or technology. They can be individuals, groups, and organizations responsible for the management and/or use of water resources and technologies, such as farmers, scientists, state and federal government, research institutes, private companies, and advocacy commissions. The fourth element stored the thematic content of the documents. This included the issues or topics about water, such as water quantity, quality and the ecosystem.

The fifth element was about the perspective/tone expressed in texts. For perspectives, two dimensions were coded to reflect people’s value inclination regarding certain water issues: “environmental sustainability oriented” or “economic development oriented”. Perspectives expressed as addressing economic development such as increasing water for irrigation were coded as “economic oriented”; whereas those expressing concerns for water over-allocation and degraded ecosystems were coded as “environmental sustainability oriented”. For sematic tone, three dimensions were coded to reflect people’s willingness regarding certain water issues: positive (supportive), negative (oppositive) and neutral.

#### Content coding

Manual content analysis was conducted to transform the unstructured textual information from each document into a structured format. Given the highly varied formats of different data sources, we believed that human coders were more alert to the implied and contextual arguments in text. All coders were professionally trained academics in the water resource governance field with English proficiency. Coders responsible for coding Chinese or Spanish documents were also proficient in the corresponding languages. They were trained before coding to be familiar with the coding framework, and disagreements in the coder’s interpretation of articles and classifications were resolved through discussions to further improve coding consistency.

We adopted an inductive coding approach. Coders conducted coding according to the “5 W” elements in Table 4 initially. As coding proceeded, the “5 W” elements that were not present in text were removed (e.g., the “What perspective/tone” element for technology and policy) and the contents were iteratively added and summarised as different thematic groups under each element to ensure all contents were included. All coders independently coded all documents. Inter-coder reliability tests were then conducted to calculate the rate of coding agreement using the Krippendorff’s alpha^25^ to quantify the consistency of coding. More details are outlined in the Validation section below.

## Data Records

The datasets are stored in the Harvard Dataverse^56^. The “Data summary-final-v2.xslx” file stores the actors, themes, tones, temporal and spatial scales of each newspaper article, technology, and policy document. The Excel file contains 10 sheets in total, with the first being the “Content list” with links to the corresponding datasets, and the following sheets documented data for each of the following:

- “1. AUS water news”: Evolution of societal values on water in Australia;
- “2. CHN water news”: Evolution of societal values on water in China;
- “3. International TGD news”: The international public’s opinions on the Three Gorges Dam (TGD) in China
- “4. QLD flood news”: The public’s opinions changes between the two large floodings in the Brisbane River, Australia
- “5. MDB submission”: Stakeholders’ opinions on the water reforms in the Murray-Darling Basin (MDB) in Australia
- “6. Ancient tech”: Water and agricultural technologies in ancient China
- “7. River patent”: Global river-related patents
- “8. VIC water act”: Water regulations in Victoria, Australia
- “9. Chile water act”: Water regulations in Chile

The variables included are explained below and can be referred to the Supplementary Information for more specific details:

1. “When” / Time: the time of publication;
2. “Where” / Location: the spatial location in the document;
3. “Who” / Participant or organization: the individuals and organizations involved in the document;
4. “What”: the themes of the document coded according to the coding framework;
5. “What perspective or tone”: whether there is “economic development-oriented” or “environmental sustainability-oriented” perspectives; and/or positive, negative or neutral sentiment expressed in text.

## Technical Validation

The validations of data were conducted during the content coding processes, by ensuring that all qualified coders understood and followed the set rules throughout the coding processes. All coders were first trained before coding. Each document was first read and coded by two coders. They were asked to code all documents independently and a third coder with similar background was asked to code 50 randomly selected documents to check the consistency of coding. The disagreements in the coders’ interpretations of articles and classifications during the pilot coding were resolved through discussions. It is recognised that manual coding by human coders is laborious and time-consuming. On average, coding for each dataset required 12–24 months for the full coding process (including iterative coding and validation), varied depending on the data size.

**Fig. 2:**
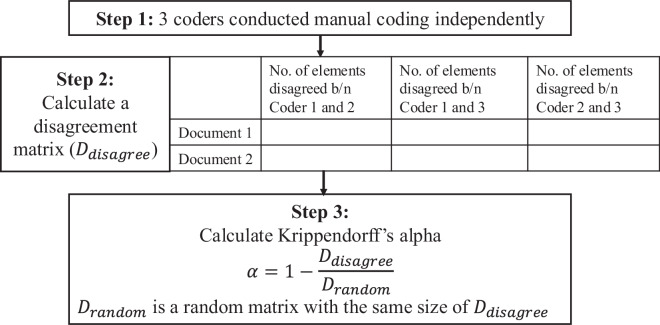
Workflow of calculating the Krippendorff’s alpha.

The level of agreements among coders was measured by the Krippendorff’s alpha^25^, which was calculated below (Fig. 2):

- All coders independently coded the contents for each document into each of the available elements defined in Table 4;
- Constructed a disagreement matrix, with rows representing the documents and columns representing the pairs of coders (N = 3). Each cell represented the disagreement (d = 1) and agreement (d = 0). The total disagreement across all coders and elements was calculated as:
  $${D}_{{disagree}}=\frac{\sum d}{N}$$
- Calculated the Krippendorff’s alpha as:

$$\alpha =1-\frac{{D}_{{disagree}}}{{D}_{{random}}}$$

where *D*_*random*_ is a random matrix with the same size of the disagreement matrix, which serves as the expected agreement by chance.

The $\alpha =1$ indicates perfect agreement among coders, lower $\alpha$ represents lower coding consistency. For initial contents coded having Krippendorff’s alpha below 80%, any disagreement and ambiguity about the coded contents were thoroughly discussed and clarified among coders. These contents were then re-coded and the updated coding principles were then applied to the following contents. This process was applied iteratively to ensure all contents coded had Krippendorff’s alpha greater than 80% to pass the inter-coder reliability tests. After this iterative coding process, the final average Krippendorff’s alpha for coding the nine datasets was 86.2%. This is well above 80% as recommended by Poindexter and McCombs^57^ indicating a high level of inter-coder reliability.

## Supplementary information

Supplementary information

## Data Availability

The datasets generated in this study are available in the Harvard Dataverse repository, 10.7910/DVN/PEENPN.

## Code availability

No custom code was used to generate or process the data described in the manuscript.
