# Supplementary material for: Quantitative datasets of societal value, technology and policy for human-water system modelling
Source: Sci Data. 2025 Sep 1;12:1528. doi: 10.1038/s41597-025-05885-x (PMC12402240; doi:10.1038/s41597-025-05885-x)
Supplement: Supplementary file 1 — Supplementary information [file 41597_2025_5885_MOESM1_ESM.docx]

Title: Quantitative dataset of societal value, technology and policy for human-water system modelling

Supplementary Information:

Detailed data information for each dataset.

Table of Contents

[1. Evolution of societal value on water in Australia 1](#_Toc205967193)

[2. Evolution of societal value on water in China 3](#_Toc205967194)

[3. The international public’s opinions on Three Gorges Dam in China 4](#_Toc205967195)

[4. The public’s opinions changes between the two large floodings in Brisbane River, Australia 5](#_Toc205967196)

[5. Stakeholders’ opinion on the water reform in the Murray Darling Basin in Australia 6](#_Toc205967197)

[6. Water and agricultural technologies in ancient China 8](#_Toc205967198)

[7. Global river-related patents 10](#_Toc205967199)

[8. Water regulations in Victoria, Australia 12](#_Toc205967200)

[9. Water regulations in Chile 13](#_Toc205967201)

# **Evolution of societal value on water in Australia**

**Reference articles: (Wei et al., 2023; Wei et al., 2017; Wei et al., 2015)**

Wei, J., Wei, Y., Tian, F., Xiong, Y., & Hu, H. (2023). Transition in the societal value and governance of water resources in Australia and China. Humanities and Social Sciences Communications, 10(1), 359. <https://doi.org/10.1057/s41599-023-01857-x>

Wei, J., Wei, Y., & Western, A. (2017). Evolution of the societal value of water resources for economic development versus environmental sustainability in Australia from 1843 to 2011. Global Environmental Change, 42, 82-92. <https://doi.org/10.1016/j.gloenvcha.2016.12.005>

Wei, J., Wei, Y., Western, A. et al. (2015). Evolution of newspaper coverage of water issues in Australia during 1843–2011. AMBIO 44, 319–331. <https://doi.org/10.1007/s13280-014-0571-2>

**Time:** 1843 – 2020

**Data source:** News published by the Sydney Morning Hearld from Trove (1843–1954), The Sydney Morning Herald’s archives (1955–1986) and Factiva (1987–2020) data sources.

**Search keyword:** “water”

**Dataset size:** 3644

**Variables coded:**

Table S1. Coding table for societal value on water in Australia.

| **Variables** | **Description** |
| --- | --- |
| Headline | Headline of the article |
| Publication date / When | Day, month, and year on the newspaper issue; week type (data sampling) |
| Location / Where | Country, Town/Suburb, State/Province |
| Institution / Who | Government (federal, state, municipal), authorities, NGOs, industry, research institutes, individuals etc. |
| Theme / What | - **Water for urban use:** articles addressing urban demand including residential water supply and sewerage, urban water management including water consumption, water metering, and waste water management - **Water for industry:** water for mining and other industry; - **Water for agriculture:** irrigation and agricultural water conservation; - **Water for the environment:** river health, environmental degradation, environmental flow; - **Water storage and river regulation:** water storage, dam construction, river regulation, river management; - **Water policy reform and integrated management:** water pricing, water trading, water rights, policy reforms/initiatives, integrated water management, water information; - **Water quality and health:** water quality, water pollution, sanitation; - **Alternative water supply:** water recycling and re-use, rainwater harvesting, recovery of urban runoff, desalination; - **Natural hazards:** drought, flooding, climate change, bushfires; - **Recreational use:** water-based recreation, creation of water-related recreation landscape, management of water-related recreation resources; - **Exploration for water sources:** only appeared in earlier stages of European settlement, such as looking for waterholes, exploring Artesian water and other water sources for supply purposes, excursion to river reaches, etc. - **Water communication :** using rivers for navigation and communication. |
| Tone / What effect | Tone for the content:   - Environmental-oriented - Economic-oriented   If there was more than one theme in an article, code tone corresponded to each theme. One theme has its own individual tone. |

# **Evolution of societal value on water in China**

**Reference articles: (Wei et al., 2023; Xiong et al., 2016)**

Xiong, Y., Wei, Y., Zhang, Z., & Wei, J. (2016). Evolution of China's water issues as framed in Chinese mainstream newspaper. Ambio, 45(2), 241-253. <https://doi.org/10.1007/s13280-015-0716-y>

Wei, J., Wei, Y., Tian, F., Xiong, Y., & Hu, H. (2023). Transition in the societal value and governance of water resources in Australia and China. Humanities and Social Sciences Communications, 10(1), 359. <https://doi.org/10.1057/s41599-023-01857-x>

**Time:** 1946 – 2017

**Data source:** News published by The People’s Daily from the Factiva data source.

**Search keywords:** “water” in Chinese

**Dataset size:** 2059

**Variables coded:**

Table S2. Coding table for societal value on water in China.

| **Variables** | **Description** |
| --- | --- |
| Headline | Headline of the article |
| Publication date / When | Day, month, and year on the newspaper issue; week type (data sampling) |
| Location / Where | Country, Town/Suburb, State/Province |
| Institution / Who | National government agencies, river basin management agencies, water engineering management agencies, local water management agencies, local environmental protection agencies, other local agencies, scientific and social organizations, enterprises, and others. |
| Theme / What | - Flood control and drought relief - Irrigation and drainage - Urban and rural water supply - Water resources management - Water engineering construction - Water quality management - Water resources protection - Water saving - Education, science and culture |
| Tone / What effect | Tone for the content:   - Environmental-oriented - Economic-oriented   If there was more than one theme in an article, code tone corresponded to each theme. |

# **The international public’s opinions on Three Gorges Dam in China**

**Reference article: (Wu et al., 2018)**

Wu, S., Huang, S., Wei, Y., Duffield, C., Tang, W., Zhao, Y., & Zheng, H. (2018). A longitudinal analysis on the perspectives of major world newspapers on the Three Gorges Dam project during 1982–2015. Water Science and Technology: Water Supply, 18(1), 94-107. <https://doi.org/10.2166/ws.2017.088>

**Time:** 1982-2015

**Data source:** 8 newspapers published outside China to focus on the international perspective of the Three Gorges Dam (TGD) in China from the Factiva data source:

- UK: The Times, The Guardian
- USA: The New York Times, The Washington Post
- Australia: The Australian, The Sydney Morning Herald
- Singapore: Lianhe Zaobao, The Straits Times

**Search keywords:** “Three Gorges”, “Three Gorges Dam”, “Three Gorges Project”, “Three Gorges Construction”, and “Three Gorges Corporations” in English

**Dataset size:** 267

**Variables coded:**

Table S3. Coding table for public opinions regarding the Three Gorges Dams in China.

| **Variables** | **Description** |
| --- | --- |
| Headline | Headline of the article |
| Publication name | Name of the newspaper publisher |
| Publication date / When | Day, month, and year on the newspaper issue; week type (data sampling) |
| Location / Where | Country, Town/Suburb, State/Province |
| Institution / Who | Government (federal, state, municipal), authorities, NGOs, industry, research institutes, individuals etc. |
| Theme / What | - **Social impact:** the societal impacts of the project (e.g., displacement) - **Environment impact:** the environmental impacts of the project (e.g., erosion) - **Organization management:** if the project was considered successful - **Cost:** if the project was cost effective and under budget - **Time:** if the project was conducted/finished on time - **Quality:** if the project was conducted with quality - **Benefit:** the types of benefits the project provided (e.g., flood control, electricity generations and navigation) - **Risk:** if there were safety issues related to the project |
| Tone / What effect | Tone for the content:   - Positive - Negative - Neutral   If there was more than one theme in an article, code tone corresponded to each theme. |

# **The public’s opinions changes between the two large floodings in Brisbane River, Australia**

**Time:** 2011-2022, covering two of Queensland's most significant flood events in January 2011 and February 2022, along with 21 additional floods occurred between 2012 to 2021.

**Data source:** 262 national newspapers, state/territory, regional and suburban newspapers published in Australia in both online and print formats from the Factiva data source.

**Search keywords:** All keywords related to 'Queensland Flood' or 'Brisbane Flood', as summarised in Table S4.

Table S4. Keyword selection for collecting public opinions on the flooding in Brisbane River, Australia.

| Search requirements | Keyword search |
| --- | --- |
| Must include the following words | 'Queensland Flood' or 'Brisbane Flood' |
| Include at least one of the following words related to flooding | 'overflow', 'storm', 'storm surge', ‘rain’ or 'flash flood', 'deluge', 'inundation', 'submersion', 'waterlogging', ‘overwhelm’, ‘drown’ or ‘100-year flood’ |
| Include at least one of the following words related to flood disaster and/or response | ‘evacuation’, ‘emergency response’, ‘flood impact’, ‘disease outbreaks’, ‘rescue’, ’reconstruction’ |
| Include at least one of the following words related to flood management | ‘dam’, ‘flood insurance’, ‘urban planning’, ‘flood mitigation’, ‘drainage systems’, ‘release water’, ‘climate adaptation’, ‘structural measures’, ‘levees’, ‘flood risk management’ |

**Dataset size:** 11033

**Variables coded:**

Table S5. Coding table for public opinions on the flooding in Brisbane River, Australia.

| **Variables** | **Description** |
| --- | --- |
| Publication date / When | Day, month, and year on the newspaper issue |
| Location / Where | Local Government Areas where the reported activity occurred |
| Participant / Who | Government, Public sector, Private sector, Flood management team, Media, NGOs |
| Theme / What | - Warning and forecast - Flood preparedness - Disaster relief and response coordination - Reconstruction and repair - Recovery - Flood damage or impacts - Insurance - Donation - Flood risk management - Flood event memory |
| Tone / What effect | Tone for the content:   - Positive - Negative - Neutral   If there was more than one theme in an article, code tone corresponded to each theme. |

# **Stakeholders’ opinion on the water reform in the Murray Darling Basin in Australia**

**Reference articles: (Hong et al., 2024)**

Hong, P., Wei, Y., Bouckaert, F., Johnston, K., & Head, B. (2024). Assessing stakeholder structure in water governance in the Murray-Darling Basin, a public submission perspective. Environmental Science & Policy, 156, 103746. <https://doi.org/10.1016/j.envsci.2024.103746>

**Time:** 2015, 2017, 2019.

**Data source:** Public submission repositories hosted by the Parliament of Australia, the Murray-Darling Basin Authority, and the New South Wales Department of Planning, Industry and Environment, as summarised in Table S6.

Table S6. Public repositories hosting public submissions on the water reform in the Murray-Darling Basin in Australia.

| **Receiving Entity / Data Sources** | **Policy initiative** | **Year** | **Explanation** |
| --- | --- | --- | --- |
| Parliament of Australia (APH) | Murray-Darling Basin Plan (MDBP) | 2015 | Investigate the effects of the Murray-Darling Basin Plan on regional communities. |
| Parliament of Australia (APH) | The integrity of the water market in the Murray-Darling Basin (WM) | 2017 | Address theft and corruption allegations in the Murray-Darling Basin and consequent actions by member States and the use of Commonwealth environmental water for irrigation. |
| Murray-Darling Basin Authority (MDBA) | Basin-wide environmental watering Strategy (EW) | 2019 | Provide support for environmental watering at a Basin scale and over the long term, improvements and expectations for water recovery for the Basin, and measures to improve flows in the system; and complement long-term watering plans for each Water Resource Plan area. |
| NSW Dept. Planning, Industry and Environment (NSW) | State Water Resource Plans (WRP) | 2019 | Meet requirements of the Basin Plan, and address local requirements in water management Included most sub-catchments in NSW (Attachment [Table 1](https://www.sciencedirect.com/science/article/pii/S1462901124000807#tbl0005)). |

**Dataset size:** 801

**Variables coded:**

Table S7. Coding table for public opinions on the water reform in the Murray-Darling Basin in Australia.

| **Variables** | **Description** |
| --- | --- |
| Policy initiatives / Where and When | - Murray-Darling Basin Plan (MDBP): 2015 - The integrity of the water market in the Murray-Darling Basin (WM): 2017 - Basin-wide environmental watering strategy (EW): 2019 - State Water Resource Plans (WRP): 2019 |
| Institution / Who | State Government, Local Government, Agriculture/Landowner, Irrigation/Water Supplier, Environmental Advocacy, Indigenous individuals/groups, Catchment Management Authority, National Resource Management Advisory/Consultancy |
| Theme / What | - **Implementation:**  Sufficiency of scientific research; Stakeholder engagement and communication efforts; Building water infrastructure - **Socio-ecological benefits**: Efforts to accommodate Indigenous communities; Environmental watering efforts; Current efforts to improve community wellbeing - **Governance:** Basin Plan execution and management; Implementation of water market regulation; Overall degree of government oversight accountability |
| Tone / What effect | Tone for the content:   - Positive/Satisfied - Negative/Dissatisfied - Neutral   If there was more than one theme in an article, code tone corresponded to each theme. |

# **Water and agricultural technologies in ancient China**

**Reference articles: (Wu et al., 2020a; Wu et al., 2019, 2020b)**

Wu, S., Wei, Y., Head, B., Zhao, Y., & Hanna, S. (2019). The development of ancient Chinese agricultural and water technology from 8000 BC to 1911 AD. Palgrave Communications, 5(1), 77. <https://doi.org/10.1057/s41599-019-0282-1>

Wu, S., Wei, Y., Head, B., & Hanna, S. (2020a). Measuring the Structure of a Technology System for Directing Technological Transition. Global Challenges, 5(2), 2000073. <https://doi.org/10.1002/gch2.202000073>

Wu, S., Wei, Y., Head, B., Zhao, Y., & Hanna, S. (2020). Using a process-based model to understand dynamics of Chinese agricultural and water technology development from 8000 bc to 1911 ad. Ambio. <https://doi.org/10.1007/s13280-020-01424-7>

**Time:** 8000 BC – 1912 AD, divided into 8 periods based on dynastic cycling.

**Data source:**

The three historical encyclopedias used as the data sources are summarized in Table S8.

Table S8. Data sources for water and agricultural technologies in ancient China.

| **Data sources** | **Focuses** |
| --- | --- |
| The Development History of Chinese Agriculture (Yan et al., 1993)  Publisher: Tianjing Science and Technology Publishing, China  (458 pages) | Focused on technologies using socio-economic and political contexts, structured in historical periods. |
| The History of Chinese Agricultural Technologies (Liang, 1989)  Publisher: Agricultural Publishing, China  (648 pages) | Focused on different categories of technologies, structured in historical periods. |
| The History of Science and Technology in China – The Agriculture Volume (Dong et al., 2000)  Publisher: Science Publishing, China  (883 pages) | Focused on the development of agriculture as a discipline, structured according to theories, artificial tools, and the philosophy of agricultural technology. |

**Dataset size:** 1337 technologies, 5 categories (agricultural theory, engineering, practices, protection, crops)

**Variables coded:**

Table S9. Coding table for water and agricultural technologies in ancient China.

| **Variables** | **Description** |
| --- | --- |
| Time of invention or application / When | Divided into 8 historical periods based on dynastic cycling and the characteristics of societal development:   - Neolithic: 8000 BC – 2000 BC (Primitive society) - XSZ: 2000 BC – 771 BC (Slavery society) - CQZG: 771 BC – 221 BC (Feudal society) - QH: 221 BC – 220 AD (Feudal society) - WJ: 220 AD – 581 AD (Pre-bureaucratic feudal society) - ST: 581 AD – 960 AD (Developing-bureaucratic feudal society) - SY: 960 AD – 1380 AD (Developing-bureaucratic feudal society) - MQ: 1368 AD – 1911 AD (Bureaucratic feudal society) |
| Location / Where | Divided into 6 spatial regions based on river basin boundaries, as agriculture relied on rivers to develop:   - The Yellow River Region - The Yangtze River Region - The North-eastern Region - The North-western Region - The South-eastern Region - The South-western Region   To alleviate the difficulty in clarifying the geographical ambiguities, the data sources have mapped ancient locations and their ancient names with modern locations and administrative boundaries. |
| Participants / Who | Specifically named people (e.g., agricultural expert Xu Guangqi), Position-based individuals and organizations (e.g., government officials, farmers) involved related to the invention, implementation and action related to technologies |
| Name & Type of technology / What | - **Agricultural theory:** Fertilisation science, Soil science, Agricultural meteorology, Agricultural biology, Farmland management - **Agricultural engineering:** Power sources, Tools, Irrigation infrastructure - **Organization management:** if the project was considered successful - **Agricultural practices:** Crop cultivation, Crop resource management, Breeding, Furrowing, Planting & sowing, Field cultivation, Harvest & storage - **Agricultural protection:** Natural disaster prevention, Bio-physical protection, Chemical protection - **Agricultural crops:** Food crop, Cash crop |

# **Global river-related patents**

**Reference articles: (Gan et al., 2024a, 2024b)**

Gan, L., Wei, Y., & Wu, S. (2024a). Evolution of water technology from a structural perspective. Frontiers in Environmental Science, 12. <https://doi.org/10.3389/fenvs.2024.1447120>

Gan, L., Wei, Y., & Wu, S. (2024b). Using the Evolution of a River Technology System to Compare Classification-Based and Citation-Based Technology Networks. Water, 16(19), 2856. <https://doi.org/10.3390/w16192856>

**Time:** 1900-2020

**Data source:**

Patentscope database from the World Intellectual Property Organization (WIPO) (<https://www.wipo.int/patentscope/en/>).

**Search keywords:** “river”

**Dataset size:** 40303

**Variables coded:**

Table S10. Coding table for global water related patents.

| **Variables** | **Description** |
| --- | --- |
| Patent application date / When | The year of patent application |
| Location / Where | The country of patent application |
| Applicant / Who | Individuals or entities that filed the patents applications. |
| Name & Classification of the patent / What | - **Water supply:** All patents related to water collection, water quality treatment (including water pollution and wastewater treatments), domestic and industrial supply of water. Including the following IPC codes: A23L, A47J, A61C, A61G, A61L, A62D, B01D, B01F, B01J, B01L, B02C, B06B, B08B, B09B, B09C, B21F, B25H, B25J, B28B, B28D, B29B, B29C, B30B, B32B, B64C, B64D, B65D, B65F, B65H, B66B, C01B, C01C, C01D, C01F, C01G, C02F, C03B, C03C, C07C, C07D, C07F, C08F, C08J, C08L, C09D, C10B, C10C, C11D, C12F, C12M, C12N, C12P, C14C, C22C, C23C, C23F, C23G, C25D, C30B, D04H, D07B, E01C, E01D, E01F, E01H, E02C, E02D, E02F, E03B, E03C, E03F, E04B, E04C, E04D, E04F, E04G, E04H, E21B, E21F, F04D, F15B, F16F, F16L, F16M, F17D, F22D, F23G, F23J, F24S, F24V, F27D - **Water demand:** All patents related to domestic and municipal (including recreational use), agricultural (including horticulture, animal husbandry and fishing), industrial (including transportation, energy, material, manufacturing and construction) water use. Including the following IPC codes: A01B, A01C, A01D, A01G, A01K, A01M, A01N, A22B, A22C, A23B, A23K, A23N, A43B, A45B, A45C, A45F, A47C, A47K, A61H, A61K, A62B, A62C, A63B, A63C, A63F, A63G, A63H, A63J, B03B, B03C, B03D, B04B, B04C, B05B, B07B, B23B, B23K, B23P, B24B, B24C, B25B, B27K, B28C, B44C, B60B, B60F, B60G, B60J, B60L, B60M, B60P, B60R, B60S, B60V, B60W, B61B, B61L, B62B, B62D, B63B, B63C, B63G, B63H, B63J, B64B, B65B, B65G, B66C, B66D, B66F, B67D, C04B, C05F, C05G, C08G, C09K, C10G, C10J, C10L, C10M, C12Q, C22B, C25B, C25C, D02G, D03D, D04B, D06B, D06F, D06M, D06P, E03D, E05F, E06B, E06C, E21C, E21D, F01B, F01D, F01K, F01P, F02B, F02C, F02G, F02M, F02N, F03B, F03C, F03D, F03G, F04B, F04C, F04F, F16B, F16C, F16D, F16H, F16K, F16N, F17C, F21K, F21L, F21S, F21V, F24D, F24F, F24H, F24T, F25B, F25C, F25D, F26B, F28B, F28C, F28D, F28F, F28G, F42B, F42D, G02B, G06M, G08G, G09F, G21C, G21D, H01B, H01L, H01M, H01Q, H01R, H02K, H02N, H02P, H02S, H04B, H05B. - **Water management:** All patents related to hydraulic engineering, monitoring and measuring river conditions. Including the following IPC codes: E02B, F15C, F15D, F22B, G01B, G01C, G01D, G01F, G01G, G01H, G01J, G01K, G01L, G01M, G01N, G01P, G01R, G01S, G01T, G01V, G01W, G05B, G05D, G06F, G06G, G06K, G06N, G06Q, G06T, G07C, G08B, G08C, G09B, G10L, G16C, G16Z, G21F, H01F, H01H, H01T, H02B, H02G, H02J, H03B, H03F, H03K, H04H, H04L, H04M, H04N, H04Q, H04W, H05F, H05K   **IPC classifications**  A: Human Necessities  B: Performing operations; Transporting separating; Mixing  C: Chemistry; Metallurgy  D: Textiles; Paper  E: Fixed constructions  F: Mechanical engineering; Lighting; Heating; Weapons; Blasting  G: Physics  H: Electricity |

# **Water regulations in Victoria, Australia**

**Reference article: (Werdiningtyas et al., 2020)**

Werdiningtyas, R., Wei, Y., & Western, A. W. (2020). The evolution of policy instruments used in water, land and environmental governances in Victoria, Australia from 1860–2016. Environmental Science & Policy, 112, 348-360. <https://doi.org/10.1016/j.envsci.2020.06.012>

**Time:** 1862 – 2016

**Data source:** Australasian Legal Information Institute (AUSTLII) (<https://www.austlii.edu.au/>)

**Search keywords:** “water”, “land”, “environment”; then “river”, “catchment”, “irrigation”, “flood”, “drainage”, “crown”, and “forest” in English

**Dataset size:** 425 acts

**Variables coded:**

Table S11. Coding table for water regulations in Victoria, Australia.

| **Variables** | **Description** |
| --- | --- |
| Publication date / When | The year of act publication |
| Related locations / Where | The State of Victoria, Australia |
| Entities involved / Who | Relevant actors involved, including public infrastructure providers (PIP), resource users (RU) and the resources (R) they governed at the state, municipal, local (ecoregional), and individual levels. |
| Type of policy instrument / What | The type of policy instruments implemented:   - **Procedural:**  Report/Plan (defines the presentation of information), Boundary (defines the jurisdiction of governance), Institutional framework (defines the mechanisms of information distribution among actors) - **Substantive**: Rate/Charge (sets the costs associated with resource uses), License (grants permits for resource users), Rule (regulates how resources can be used) |

# **Water regulations in Chile**

**Reference articles: (Genova et al., 2023; Genova et al., 2022)**

Genova, P., & Wei, Y. (2023). A socio-hydrological model for assessing water resource allocation and water environmental regulations in the Maipo River basin. Journal of Hydrology, 617, 129159. <https://doi.org/10.1016/j.jhydrol.2023.129159>

Genova, P., Wei, Y., & Olivares, M. A. (2022). Evolution of water environmental regulations in Chile since 1900. Water Policy, 24(8), 1306-1324. <https://doi.org/10.2166/wp.2022.053>

**Time:** 1900 – 2019

**Data source:** Ley Chile ([www.LeyChile.cl](http://www.LeyChile.cl))

**Search keywords:** “water”, “environment” and the derived words related to freshwater environment protection (in Spanish), and relevant theme filters in the search portal of the web: "agua" (water), "ambiento" (environment), "río" (river), "cuenca" (basin), "forestal" (forest).

**Dataset size:** 295 legal frameworks (Laws, DL, DFL, Decrees, and Resolutions) related to water environment protection. Each one included one or several regulations, thus over 1,400 regulations were coded.

**Variables coded:**

Table S12. Coding table for water regulations in Victoria, Australia.

| **Variables** | **Description** |
| --- | --- |
| Publication date / When | Year when the regulation was promulgated, repealed or in-force and repealed year |
| Related locations / Where | Basin(s) where it was applied, and where the regulation was applicable: all national territory, or specific region/local area. |
| Entities involved / Who | Relevant actors involved, including public infrastructure providers (PIP), resource users (RU) and the resources (R) they governed at the state, municipal, local (ecoregional), and individual levels. |
| Theme & Type of policy instruments / What | The regulations were categorized into the following seven themes:   - Surface water quality - Surface water quantity - Groundwater quality - Groundwater quantity - Terrestrial ecosystem and biodiversity - Aquatic ecosystem and biodiversity - Integrated themes (included all the themes mentioned above).   Under each theme, the regulations were categorized into nine types:   - Prohibition restrictions and obligations (PRO) - Funds - Information - Inspections - Plans to protect or restore - Environmental impact assessment and mitigation plans (EIA) - Sanctions - Protected areas - Plans for protected areas |

**References:**

Dong, K. Z., & Fan, C. Y. (2000). *The History of Science and Technology in China - The Agriculture Chapter* (J. Lu, Ed.). Science Publisher.

Gan, L., Wei, Y., & Wu, S. (2024a). Evolution of water technology from a structural perspective [Original Research]. *Frontiers in Environmental Science*, *12*. <https://doi.org/10.3389/fenvs.2024.1447120>

Gan, L., Wei, Y., & Wu, S. (2024b). Using the Evolution of a River Technology System to Compare Classification-Based and Citation-Based Technology Networks. *Water*, *16*(19), 2856. <https://www.mdpi.com/2073-4441/16/19/2856>

Genova, P., & Wei, Y. (2023). A socio-hydrological model for assessing water resource allocation and water environmental regulations in the Maipo River basin. *Journal of Hydrology*, *617*, 129159. https://doi.org/10.1016/j.jhydrol.2023.129159

Genova, P., Wei, Y., & Olivares, M. A. (2022). Evolution of water environmental regulations in Chile since 1900. *Water Policy*, *24*(8), 1306-1324. <https://doi.org/10.2166/wp.2022.053>

Hong, P., Wei, Y., Bouckaert, F., Johnston, K., & Head, B. (2024). Assessing stakeholder structure in water governance in the Murray-Darling Basin, a public submission perspective. *Environmental Science & Policy*, *156*, 103746. https://doi.org/10.1016/j.envsci.2024.103746

Liang, J. (1989). *The History of Chinese Agricultural Technologies*. Agricultural Publisher.

Wei, J., Wei, Y., Tian, F., Xiong, Y., & Hu, H. (2023). Transition in the societal value and governance of water resources in Australia and China. *Humanities and Social Sciences Communications*, *10*(1), 359. <https://doi.org/10.1057/s41599-023-01857-x>

Wei, J., Wei, Y., & Western, A. (2017). Evolution of the societal value of water resources for economic development versus environmental sustainability in Australia from 1843 to 2011. *Global Environmental Change*, *42*, 82-92. <https://doi.org/10.1016/j.gloenvcha.2016.12.005>

Wei, J., Wei, Y., Western, A., Skinner, D., & Lyle, C. (2015). Evolution of newspaper coverage of water issues in Australia during 1843-2011. *Ambio*, *44*(4), 319-331. <https://doi.org/10.1007/s13280-014-0571-2>

Werdiningtyas, R., Wei, Y., & Western, A. W. (2020). The evolution of policy instruments used in water, land and environmental governances in Victoria, Australia from 1860–2016. *Environmental Science & Policy*, *112*, 348-360. <https://doi.org/10.1016/j.envsci.2020.06.012>

Wu, S., Huang, S., Wei, Y., Duffield, C., Tang, W., Zhao, Y., & Zheng, H. (2018). A longitudinal analysis on the perspectives of major world newspapers on the Three Gorges Dam project during 1982–2015. *Water Science and Technology: Water Supply*, *18*(1), 94-107. <https://doi.org/10.2166/ws.2017.088>

Wu, S., Wei, Y., Head, B., & Hanna, S. (2020a). Measuring the structure of a technology system for directing technological transition. *Global Challenges*, *5*(2), 2000073. <https://doi.org/10.1002/gch2.202000073>

Wu, S., Wei, Y., Head, B., Zhao, Y., & Hanna, S. (2019). The development of ancient Chinese agricultural and water technology from 8000 BC to 1911 AD. *Palgrave Communications*, *5*(1), 77. <https://doi.org/10.1057/s41599-019-0282-1>

Wu, S., Wei, Y., Head, B., Zhao, Y., & Hanna, S. (2020b). Using a process-based model to understand dynamics of Chinese agricultural and water technology development from 8000 bc to 1911 ad. *Ambio*. <https://doi.org/10.1007/s13280-020-01424-7>

Xiong, Y., Wei, Y., Zhang, Z., & Wei, J. (2016). Evolution of China's water issues as framed in Chinese mainstream newspaper. *Ambio*, *45*(2), 241-253. <https://doi.org/10.1007/s13280-015-0716-y>

Yan, W. Y., & Yin, Y. H. (1993). *The Development History of Chinese Agriculture*. Tianjing Science and Technology Publisher. <https://books.google.com.au/books?id=6C0yAAAAMAAJ>
